# Functions of Two Distinct “Prolactin-Releasing Peptides” Evolved from a Common Ancestral Gene

**DOI:** 10.3389/fendo.2014.00170

**Published:** 2014-11-10

**Authors:** Tetsuya Tachibana, Tatsuya Sakamoto

**Affiliations:** ^1^Department of Agrobiological Science, Faculty of Agriculture, Ehime University, Matsuyama, Japan; ^2^Ushimado Marine Institute, Faculty of Science, Okayama University, Ushimado, Japan

**Keywords:** prolactin-releasing peptide, prolactin-releasing peptide-2, C-RFa, vertebrates, prolactin, feeding, stress

## Abstract

Prolactin-releasing peptide (PrRP) is one of the RF-amide peptides and was originally identified in the bovine hypothalamus as a stimulator of prolactin (PRL) release. Independently, another RF-amide peptide was found in Japanese crucian carp and named *Carassius*-RFa (C-RFa), which shows high homology to PrRP and stimulates PRL secretion in teleost fish. Therefore, C-RFa has been recognized as fish PrRP. However, recent work has revealed that PrRP and C-RFa in non-mammalian vertebrates are encoded by separate genes originated through duplication of an ancestral gene. Indeed, both PrRP and C-RFa are suggested to exist in teleost, amphibian, reptile, and avian species. Therefore, we propose that non-mammalian PrRP (C-RFa) be renamed PrRP2. Despite a common evolutionary origin, PrRP2 appears to be a physiological regulator of PRL, whereas this is not a consistent role for PrRP itself. Further work revealed that the biological functions of PrRP and PrRP2 are not limited solely to PRL release, because they are also neuromodulators of several hypothalamus–pituitary axes and are involved in some brain circuits related to the regulation of food intake, stress, and cardiovascular functions. However, these actions appear to be different among vertebrates. For example, central injection of PrRP inhibits feeding behavior in rodents and teleosts, while it stimulates it in chicks. Therefore, both PrRP and PrRP2 have acquired diverse actions through evolution. In this review, we integrate the burgeoning information of structures, expression profiles, and multiple biological actions of PrRP in higher vertebrates, as well as those of PrRP2 in non-mammals.

## Discovery of Prolactin-Releasing Peptides

Hinuma et al. (1) first identified a peptide, which is a ligand of the orphan 7-transmembrane receptor hGR3, by using a reverse-pharmacological technique. The peptide was named “prolactin-releasing peptide (PrRP)” because it was found to specifically promote prolactin (PRL) release from rat anterior pituitary cells *in vitro*.

Independent of the discovery of mammalian PrRP, a novel RF-amide peptide, which has a similar amino acid sequence, was isolated from the brain of the Japanese crucian carp using an intestine contracting assay (2) and named *Carassius*-RFa (C-RFa). In addition to the sequence homology, C-RFa is a strong candidate as the stimulator of PRL synthesis and release, and this peptide has been regarded as a teleost PrRP. However, some studies reported that PrRP did not show a PRL-releasing effect in mammals (3). These differences implied that PrRP and C-RFa might not be orthologous. Finally, with a synteny analysis, Lagerström et al. (4) and Wang et al. (5) found that PrRP and C-RFa originated in gene duplication from a common ancestral gene, and we propose that the widely used name of PrRP in lower vertebrate species be renamed “PrRP2.”

## Biochemistry and Molecular Biology of PrRP and PrRP2

### PrRP

**Figure 1:**
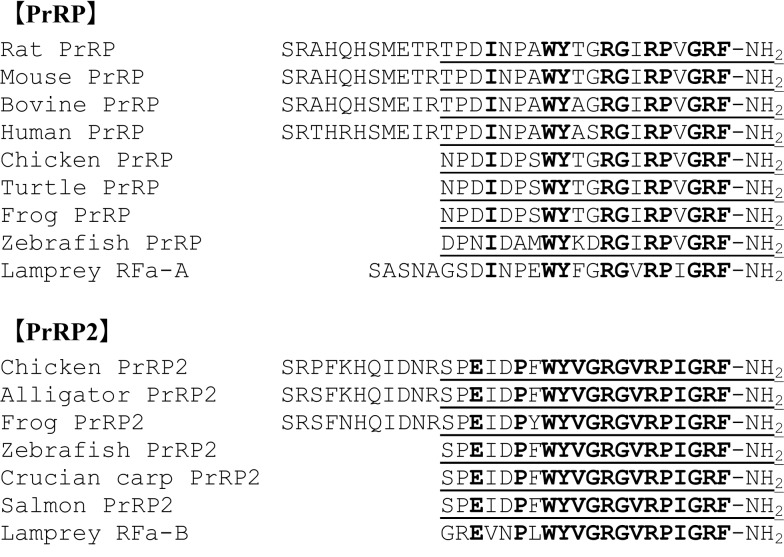
**Identified or predicted amino acid sequences of PrRP and PrRP2 in vertebrates**. Bold characters indicate conserved amino acid residues. Identified or predicted PrRP-20 or PrRP2-20 peptide sequences are underlined.

Prolactin-releasing peptide was first identified in the bovine hypothalamus (1). There are two types of PrRP in mammals: one consists of 20 amino acids (PrRP-20) and the other consists of 31 amino acids (PrRP-31) (Figure 1), with PrRP-20 being a C-terminal fragment of PrRP-31. As well as in mammals, PrRP-20 is also predicted from the cDNA sequences in some non-mammalian vertebrates, such as chicken, *Xenopus tropicalis*, and zebrafish (5), while the existence of a longer form of PrRP such as PrRP-31 has been unclear (Figure 1). These amino acid sequences of PrRP-20 are well conserved. Among mammals, rat and mouse PrRP-20 are identical and their amino acid sequence shows high homology to bovine and human counterparts. Chicken and *X. tropicalis* have the same PrRP-20, whose sequence is different from murine PrRP-20 at only three amino acids. The amino acid sequence of zebrafish PrRP-20 shows moderate homology to those of mammals, and of chicken or *X. tropicalis*. All PrRPs have the Arg-Phe-NH_2_ (RF-amide) motif at the C-terminus. The C-terminus amidation is necessary for interaction with the receptors because PrRP-20 with a non-amidated C-terminus cannot efficiently interact (1). Rat and human PrRP genes are located on chromosomes 9q36 and 2q37.3, respectively. The genomic organization and promoter function of the rat PrRP gene have been examined by Yamada et al. (6).

In non-mammalian vertebrates, the structure of the PrRP gene has not been well investigated. Since non-mammalian vertebrates have two types of PrRP (namely, PrRP and PrRP2), which is orthologous to mammalian PrRP was a problem. This problem was solved by the synteny analysis done by Wang et al. (5). Chicken, *X. tropicalis*, and zebrafish PrRP genes are located on chromosome 7, scaffold 15, and chromosome 9, respectively. These PrRP genes are surrounded by several genes such as *leucine-rich repeat interacting protein 1*, *ras-related protein Rab-17*, *melanophilin*, and *collagen type VI, alpha 3* (5). These neighboring genes (surrounding the PrRP gene) are also observed on human chromosome 2q37.3, where the PrRP gene is located. Based on PrRP genes in non-mammalian vertebrates, the amino acid sequence of their preproPrRPs was predicted and its sequence showed low-to-moderate homology to mammalian PrRP.

### PrRP2

Fujimoto et al. (2) first isolated a novel bioactive peptide from the Japanese crucian carp. Since the peptide contains an RF-amide sequence at the C-terminus, it was named *Carassius* RFamide (C-RFa). Later, C-RFa homologs were isolated from several teleosts, such as chum salmon and tilapia, and their amino acid sequences are identical to the crucian carp (7–9). This amino acid sequence shows similarity to those of mammalian PrRP-20 (Figure 1). Together with their stimulation of PRL release in teleosts (10, 11), they have been considered the teleost orthologs of PrRP-20 (12). However, a recent phylogenetic study revealed that they do not originate from the same gene as mammalian PrRP (5). It is, therefore, natural that the nomenclature should be reviewed and it would be better renamed PrRP2 (5). Sea bream and zebrafish PrRP2-20s predicted from the cDNA also show the identical sequence [Figure 1; (5, 13, 14)]. Additionally, two putative PrRP2 consisting of 37 or 36 amino acids, like PrRP-31, were predicted from the analyses of cleavage sites, but such a longer form of PrRP2 has not been identified in teleosts.

PrRP2 was also identified in chicken: both PrRP2-31, which consists of 31 amino acids and the C-terminal PrRP2-20 are present in the brain (15). In several non-mammalian vertebrates, such as *Xenopus laevis* and *X. tropicalis*, PrRP2s have been predicted from cDNA (5, 16). All the PrRP2s are expected to have RF-amide motifs at the end of their C-terminuses and their PrRP2-20s show high homology to teleost PrRP2. The amino acid sequences of teleost PrRP2 and chicken PrRP2-20 are identical (Figure 1), and the sequence of *Xenopus* shows high homology (Figure 1). In one animal species, the sequence of PrRP2 is moderately similar to that of the respective PrRP. PrRP-like RF-amide peptides are also found in sea lamprey, and named RFa-A and RFa-B (17) (Figure 1). These peptides show relatively high homology to teleost, *Xenopus*, and chicken PrRP2 but not to PrRP, suggesting that they are homologous to PrRP2. RFa-B, in particular, has an amino acid sequence similar to teleost, *Xenopus*, and chicken PrRP2s compared with RFa-A. If lamprey RFa-A and RFa-B are, respectively, orthologous to PrRP and PrRP2, these peptides originated at least from the stage of primitive vertebrates.

Chicken and *X. laevis* preproPrRP2 consist of 108 amino acids (15, 16). Chicken and *X. tropicalis* preproPrRP2, respectively, have low homology with their preproPrRP (5, 15). Their amino acid sequences suggest the occurrence of a longer form of PrRP2 such as PrRP2-31 in *X. laevis* (16) as shown in chickens. The deduced amino acid sequence of *X. laevis* PrRP2-31 shows high homology with chicken PrRP2-31 and moderate homology with mammalian PrRP-31. Although PrRP2-20 can activate PrRP-R1, PrRP-R2, and PrRP2-R in chickens (5), N-terminal 11 residue sequence of PrRP2-31 also seem to be important for activity because central injection of PrRP2-20 did not affect feeding behavior and blood constituents while PrRP2-31 stimulates feeding behavior and modified blood constituents (15, 18).

**Figure 2:**
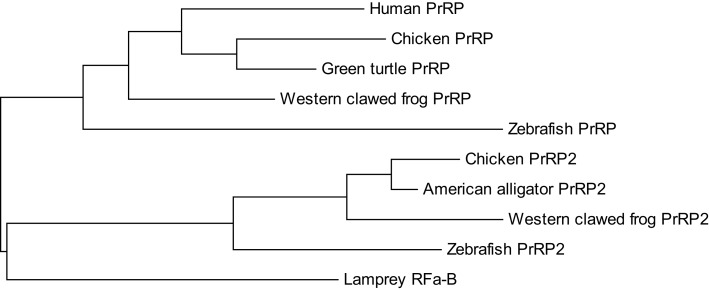
**Phylogenetic relationship between proPrRP and proPrRP2 in vertebrates**. The tree was constructed using the maximum likelihood method, plotted in MEGA6.

In contrast to non-mammalian vertebrates, PrRP2 has not been identified in mammals. PrRP2 genes are located on chromosome 3, scaffold 359, and chromosome 24 in chicken, *X. tropicalis*, and zebrafish, respectively, and genes of *myosin VIIA* and *Rab interacting protein* (*MYRIP*) exist on each chromosome or scaffold (5). The *MYRIP* gene is located on human chromosome 3, but the PrRP2 gene has not been identified. Based on a phylogenetic relationship among PrRPs and PrRP2s (Figure 2), divergence of PrRP and PrRP2 from the common ancestral gene occurred faster than their specialization in each vertebrate. It is, therefore, possible that PrRP2 might have been lost during mammalian evolution. Lagerström et al. (4) could not find PrRP or PrRP2 in any invertebrate genome, suggesting that these peptides most likely arose during the tetraploidizations.

## Receptors for PrRP and PrRP2

Hinuma et al. (1) first identified the human PrRP receptor (hGR3, PrRP-R), which is virtually identical to the orphan receptor GPR10 (19). The hGR3 receptor was expected to be a counterpart of the rat orphan receptor UHR-1 (20), sharing 89% amino acid homology. These PrRP-Rs share a slight homology with the neuropeptide Y (NPY) receptor in amino acid sequences, and a micromolar level of NPY was able to bind and inhibit completely the PrRP-induced response in cells, which express PrRP-R, suggesting that PrRP-R shares a common ancestor with NPY receptors (13). On the other hand, it is likely that PrRP binds to not only the PrRP receptor but also other receptors such as neuropeptide FF receptor-2 (21).

In non-mammalian vertebrates, Lagerström et al. (13) first identified PrRP-R. They found three PrRP-Rs (PRLHR1, PRLHR1B, and PRLHR2) in chicken, two receptors (PRLHR1 and PRLHR2) in pufferfish, and one receptor (PRLHR2) in zebrafish. Thereafter, Wang et al. (5) suggested that PRLHR1 and PRLHR1B are receptors for both PrRP and PrRP2 in chicken while PRLHR2 is a specific receptor for PrRP2 in chicken. Based on these features, the names were changed from PRLHR1 and PRLHR1B to PrRPR1 and PrRPR2, respectively, and from PRLHR2 to C-RFaR (5). As noted above, we suggest that C-RFa be renamed PrRP2, so C-RFa-R should be renamed PrRP2-R. In this review, we use the PrRP receptor terms PrRP-R1, PrRP-R2, and PrRP2-R for chicken and other non-mammalian vertebrates.

### PrRP-R

Mammalian PrRP-R belongs to the 7-transmembrane receptor (7TMR) superfamily. Radioiodinated PrRP-20 and -31 bind equally and with high affinity to these orphan 7TMRs expressed in CHO cells and HEK293 cells (1, 22, 23). Except for non-mammalian PrRP2, none of the known native ligands including RF-amide neuropeptide FF demonstrate any affinity for GPR10 (23). These studies indicate that PrRPs are specific, high affinity ligands for PrRP-R. In rats, Satoh et al. (24) suggested that there may be different subtypes of PrRP-R, which may be different from UHR-1.

The human PrRP-R gene is located on chromosome 10q26.13 and consists of two exons and one intron (25), although the entire coding region is intronless. In GH3 pituitary tumor cells or primary cultures of anterior pituitary cells from rat, the PrRP receptor appears to signal via multiple kinase pathways including mitogen-activated protein kinase (MAPK), Jun N-terminal kinase (JNK), and serine/threonine kinase (Akt/protein kinase B) to the PRL promoter; these pathways require an Ets transcription factor (26, 27). Since the Akt pathway is associated with cell survival and growth, PrRP may function not only to control PRL expression, but also to maintain PRL cell number (28).

**Figure 3:**
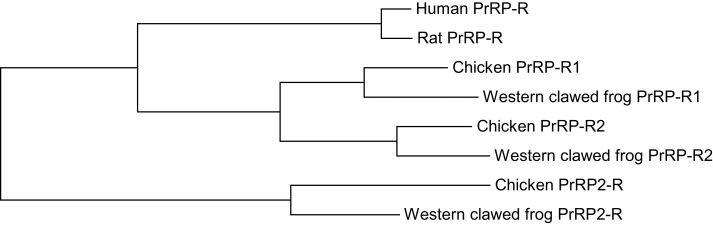
**Phylogenetic relationship between PrRP-R, PrRP-R1, PrRP-R2, and PrRP2-R in vertebrates**. The tree was constructed using the maximum likelihood method, plotted in MEGA6.

In non-mammalian vertebrates, two subtypes of the PrRP receptor, PrRP-R1 and PrRP-R2, have been predicted (5, 13). In *X. tropicalis* and chicken, the amino acid sequences of PrRP-R1 and PrRP-R2 are moderately conserved and show moderate homology with mammalian PrRP-R. The PrRP-R1 and PrRP-R2 genes are located on scaffold 605 and 40, and on chromosome 6 and 22 in chickens (5), respectively. Their surrounding genes are well conserved between human beings, chickens, and *Xenopus*. Based on a synteny analysis, PrRP-R1 but not PrRP-R2 is thought to be orthologous to mammalian PrRP-R [Figure 3; (13)]. Although the PrRP-R2 gene is expected to exist on chromosome 8 in human beings based on the surrounding genes, it has not yet been identified. Like mammalian PrRP-R, the entire coding region of cPrRPR1 is intronless while the coding region of cPrRPR2 is interrupted by an intron (5, 13). In chickens, both PrRP and PrRP2 show affinity to PrRP-R1 and PrRP-R2 to a similar extent (5). Both receptors possess conserved structural motifs, which are also well conserved within the rhodopsin family of G-protein coupled receptors. In chickens, PrRP-R1 and PrRP-R2 appear to be functionally coupled with the intracellular protein kinase A signaling pathway (5). The activation of these receptors is also expected to trigger Ca^2+^ release from intracellular stores (5).

Teleost PrRP-Rs are also predicted from their genome. For example, Watanabe and Kaneko (29) characterized PrRP-R of tilapia. This receptor may be categorized as PrRP-R2 because its amino acid sequence shows higher homology to *X. tropicalis* and chicken PrRP-R2 rather than other receptors.

### PrRP2-R

PrRP2-Rs have been identified in several non-mammalian vertebrates, such as zebrafish, *X. tropicalis*, and chickens (5, 13). Among these animals, the structures of *Xenopus* and chicken PrRP2-R have been well studied. The entire coding region of the chicken PrRP2-R gene is intronless (5), while *X. tropicalis* PrRP2-R consists of two exons and one intron as does mammalian PrRP-R. *Xenopus* and chicken PrRP2-R, respectively, consists of 364 and 361 amino acids, and show moderate homology between them. In contrast, *Xenopus* and chicken PrRP2-R also show lower homology to their PrRP-R1 and PrRP-R2, respectively.

Like PrRP-Rs, PrRP2-R belongs to the neuropeptide receptor family of the 7TMR superfamily. In chickens, stimulation of PrRP2-R also activates the intracellular protein kinase A signaling pathway. Chicken PrRP2-R is activated by both PrRP and PrRP2 *in vitro*, but PrRP2 is 150 times more potent than PrRP (5). On the other hand, chicken PrRP-Rs are similarly activated by PrRP and PrRP2. Therefore, PrRP2-R is a specific receptor for PrRP2 in chickens. This idea was supported by studies using teleost showing that Japanese crucian carp PrRP2 can also interact with human PrRP-R (30). In addition, teleost PrRP2, not mammalian PrRP, stimulates PRL release from tilapia pituitary, suggesting that PrRP2-R might be related to PRL secretion and mammalian PrRP would not be able to bind to teleost PrRP2-R (8).

Chicken, *X. tropicalis*, and zebrafish PrRP2-R genes are located on chromosome 5, scaffold 627, and chromosome 17, respectively (5). Genes surrounding the PrRP2-R gene are well conserved between vertebrates including mammals. The human PrRP2-R gene is expected to exist on chromosome 15, but it has not yet been identified as well as PrRP2 gene (5). Phylogenetic study implies that the PrRP2 and PrRP-R2 genes disappeared through the evolution of mammals.

## Distribution of PrRP, PrRP2, and Their Receptors

### PrRP and PrRP-R

Prolactin-releasing peptide is widely distributed in central and peripheral organs in mammals (Table 1). In the rat brain, PrRP nerve cell bodies are distributed in the hypothalamic dorsomedial nucleus (DMN), the nucleus of the solitary tract (NTS), and ventral and lateral reticular nuclei (VLRN) in the medulla oblongata (31–33). PrRP neurons in the VLRN and NTS are considered to be A1 and A2 noradrenergic neurons, respectively (34, 35). PrRP nerve fibers project to several brain regions, such as the bed nucleus of the stria terminalis, hypothalamic paraventricular nucleus (PVN), hypothalamic periventricular nucleus (PerVN), and basolateral amygdaloid complex (31, 36). PrRP nerve fibers in the hypothalamus appear to contact catecholaminergic, oxytocin, corticotrophin-releasing hormone (CRH), and somatostatin neurons (36–39). On the other hand, PrRP neurons do not project to the external lamina of the median eminence (33, 40), where hypophysiotropic factors of hypothalamic origin gain access to the hypophysial portal vasculature and the anterior lobe. An axonal transport study (35) also revealed that the hypothalamic PrRP neurons are not directly connected to the anterior pituitary. The distribution of PrRP in the brain was also investigated in human beings and sheep, and their distributions are basically similar to those of rats (41–43). PrRP and its mRNA have also been found in a number of peripheral tissues, including the pituitary gland, adrenal gland, lung, pancreas, and testis (41, 44) (Table 1).

**Table 1:** **Comparison of the distribution of PrRP and PrRP2 mRNA among mammals, avians, and teleosts**.

	Mammal^a^	Avian^b^		Teleost
	PrRP	PrRP	PrRP2	PrRP2
Whole brain	+	+	+	+^c^
Telencephalon	–	+	+	–^d^, +^e^
Diencephalon	+	+	+	+^d^^,^^e^
Midbrain	–	+	+	+^d^^,^^e^
Cerebellum	–	+	+	–^d^
Hindbrain	+	+	+	+^d^^,^^e^
Spinal cord	+	–	–	+^d^
Pituitary	–	+	+	+^c^^,^^d^, –^e^
Thyroid gland	+			
Heart	–	–	+	–^d^
Lung	+	+	+	
Liver	–	–	+	+^c^
Spleen	–	–	–	–^c^
Pancreas	+	–	+	
Kidney	+	–	+	–^c^^,^^d^
Gut	+	–	+	+^c^^,^^d^
Adipose tissue	–			
Skeletal muscle	–	+	+	–^c^
Testis	+	+	+	
Ovary	–	+	+	+^c^

*^a^Rat (41)*.

*^b^Chicken (5)*.

*^c^Mudskipper (12)*.

*^d^Sea bream (14)*.

*^e^Goldfish (45)*.

*+: Definite expression; –: little or nothing*.

Blank columns indicate “not examined.”

*Distribution of PrRP has not yet been investigated in teleost*.

*In rats, PrRP mRNA expression is also observed in thymus, trachea, submandibular gland, adrenal gland, and uterus (see PrRP and PrRP-R)*.

As with PrRP itself, PrRP-R-expressing neurons are widely distributed throughout the brain, including the PVN, hypothalamic preoptic area (POA), PerVN, ventrolateral hypothalamus, DMN, reticular thalamus, and area postrema (22, 33, 38). In contrast to the central nervous system, the distribution of PrRP-R seems to be sparse in the peripheral tissues. PrRP receptor mRNA has been found in the pituitary gland, adrenal gland, stomach, and femur (41).

In contrast to mammals, the distribution of PrRP in non-mammalian vertebrates has not been well investigated. Wang et al. (5) found that PrRP mRNA is expressed in both central and peripheral tissues in chickens. In the central nervous system, PrRP mRNA is present in various brain regions including the hypothalamus, but not in the spinal cord (Table 1). In the peripheral tissues, PrRP mRNA is present in the pituitary, muscle, lung, testis, and ovary. These distributions in chickens are almost similar to those of mammals.

In chickens, PrRP-R1 and PrRP-R2 mRNA are widely and similarly distributed throughout the brain as they are in mammals (5). On the other hand, their distribution in the peripheral tissues is different (5). PrRP-R1 mRNA is present in the pituitary, heart, small intestine, kidney, liver, lung, muscle, testis, ovary, and spleen. PrRP-R2 mRNA is distributed in similar tissues to PrRP-R1, but it is not present in the pituitary, liver, and spleen. The difference in distribution indicates that they are functionally differentiated in chickens. In tilapia, PrRP-R (probably PrRP-R2) mRNA is expressed in the brain, pituitary, heart, spleen, kidney, and rectum but not liver (29). The mRNA expressions of PrRP-R in the peripheral tissues are different between vertebrates, suggesting that the action of PrRP is different between chickens and mammals.

### PrRP2 and PrRP2-R

PrRP2 mRNA is distributed in the central nervous system in non-mammalian vertebrates (5, 12, 14, 45, 46) (Table 1). Further, a histological survey has been performed only in teleosts using the antisera against teleost PrRP2 whose cross-reactions with PrRP are unclear. Based on these studies, PrRP and/or PrRP2 perikarya and nerve fibers are thought to be distributed in the hypothalamus and pituitary of teleosts (7, 47). Further studies using specific antiserum for PrRP2 will clarify the actual distribution of PrRP2 in the brain of teleosts.

PrRP2 mRNA is abundantly expressed not only in the brain but also in the peripheral organs of teleosts (Table 1). In mudskippers, PrRP2 mRNA is expressed in the liver, gut, and ovary, while significant levels of expression were also detected in the skin and kidney (48). Within the cyprinid retina, PrRP2 mRNA is also abundantly expressed (46). Corresponding to the distribution of PrRP2 mRNA in the mudskipper, relatively high expression of extrapituitary PRL was observed in the liver, gut, and ovary (48). PrRP2 may stimulate PRL expression in an autocrine/paracrine manner as observed in human decidua PrRP-PRL (49).

In chickens, PrRP2 mRNA and mature peptides (PrRP2-20 and PrRP2-31) are expressed in the telencephalon, midbrain, cerebellum, hindbrain, and hypothalamus of the brain (5, 15). Messenger RNA is also expressed in the peripheral organs such as the heart, small intestine, kidney, liver, lung, muscle, ovary, testis, pituitary, and pancreas (5). PrRP2-R mRNA is also observed in similar tissues to PrRP2, but it is not expressed in the heart and pancreas.

## Biological Actions of PrRP and PrRP2

In vertebrates, it has been reported that both PrRP and PrRP2 are related to many physiological roles such as the regulation of pituitary hormone release, feeding/energy metabolism, stress response, cardiovascular regulation (50), retinal information processing (51), regulation of sleep (52), and stimulation of visceral muscle contraction (2) (Table 2). Brain PrRP is also thought to be related to the regulation of reproduction because brain PrRP mRNA expression increases during proestrus of the gonadal cycle and mid-period of pregnancy (53). In *Xenopus*, PrRP2 might be related to the regulation of metamorphosis because PrRP2 mRNA expression peaks at premetamorphosis (16). These functions have been well summarized in several reviews [for example, see Ref. (12, 54–56)]. Among them, the regulations of pituitary hormone release, feeding behavior, and stress have been well studied in both PrRP and PrRP2. In this section, we describe the current knowledge of the actions of PrRP and PrRP2 in PRL release, hypothalamus–pituitary axes, feeding/energy metabolism, and stress.

**Table 2:** **Comparison of representative actions of PrRP and PrRP2 between mammals, avians, and teleosts**.

Action	Mammal	Avian^a^		Teleost
	PrRP	PrRP	PrRP2	PrRP2
PRL release
*In vitro*	↑^b^^,^^c^, →^b^^,^^c^			↑^d^^,^^e^
*In vivo*				
Peripheral injection	↑^b^, →^f^^,^^g^		↑	↑^d^^,^^e^
Central injection	↑^b^^,^^f^, →^g^	↓	↓	
GH release
*In vitro*	↑^c^			↓^e^, →^d^
*In vivo*				
Peripheral injection			↓	↓^d^, →^e^
Central injection	↓^b^		↓	
Activation of HPA axis
Peripheral injection				→^e^^,^^h^
Central injection	↑^b^^,^^f^, →^g^	↑	→	
Food intake
Peripheral injection		→	→	↓^h^
Central injection	↓^b^^,^^i^, →^f^	↑	↑	

*^a^Chicken (15, 18, 57)*.

*^b^Rat (1, 3, 37, 38, 52, 58, 67)*.

*^c^Human being (59)*.

*^d^Rainbow trout (7)*.

*^e^Tilapia [PRL-releasing effect induced by peripheral injection was observed only in females and suppression of GH release *in vitro* was observed only in males, (8)]*.

*^f^Cattle (60, 61)*.

*^g^Sheep (43, 62)*.

*^h^Goldfish (11, 45)*.

*^i^Mouse (63)*.

*↑: Increase; ↓: Decrease; →: No change*.

Blank columns indicate “not examined.”

*Actions of PrRP have not yet been investigated in teleost*.

*In rats, PrRP is related to the releases of oxytocin, vasopressin, FSH, and LH, and the regulation of stress response, sleeping, blood pressure, and so on (see Biological Actions of PrRP and PrRP2)*.

*In Japanese crucian carp, PrRP alters somatolactin secretion and contracts the intestine (see Discovery of Prolactin-Releasing Peptides and Biological Actions of PrRP and PrRP2)*.

### Effect on PRL secretion

#### PrRP

Hinuma et al. (1) first found that PrRP stimulates PRL release from the rat pituitary adenoma-derived cell line and from primary cultures of anterior pituitary cells harvested from lactating female rats, the most sensitive model of PRL-releasing factor activity (64). This PRL-releasing activity appeared to be specific since PrRP did not affect the release of other anterior pituitary hormones such as luteinizing hormone (LH), follicle-stimulating hormone (FSH), thyroid-stimulating hormone, growth hormone (GH), and adrenocorticotropic hormone (ACTH). However, further studies revealed that the effect of PrRP on PRL secretion was inconsistent. For example, PrRP stimulated PRL release only when a higher dose was applied (65, 66) or had no effect (3). *In vivo*, intravenous (IV) injection of PrRP-31 induces PRL release dependent on the estrus cycle and estrogen in rats (67, 68). On the other hand, IV injection of PrRP has no effect on PRL secretion in cattle (69), while intracerebroventricular (ICV) injection of PrRP temporarily increases PRL secretion (60). In addition to this, neither IV nor ICV injections induce PRL secretion in female sheep (43), suggesting that PrRP is not important in stimulating PRL secretion in ruminants.

As noted above, PrRP neuronal terminals are not observed in the external layer of the median eminence, suggesting that PrRP is expected to stimulate PRL release with different mechanism(s) from ordinary hypophysiotropic hormones. The small number of PrRP nerve fibers in the posterior pituitary (36) suggests that PrRP in the posterior pituitary might be transported to the anterior pituitary through a short hypophyseal portal system. PrRP-R expressed not only in the anterior pituitary but also in the rostral and medio-basal hypothalamus and in the hypothalamic PVN suggests possible indirect pathways, which modulate PRL release, but the effects of PrRP on PRL release are not consistent among studies in rats (3, 52, 70–73). In the hypothalamic explants, PrRP-31 increases the release of vasoactive intestinal peptide (VIP) and galanin, which are known to stimulate PRL release (70). ICV injection of PrRP stimulates activity of tuberoinfundibular dopaminergic neurons, which are involved in regulation of PRL secretion (39). It is, therefore, possible that PrRP modifies the activity of dopaminergic, VIP, and galanin neurons in the hypothalamus and then stimulates PRL release. In human beings, PrRP and PrRP-R are expressed in the uterine decidua and PrRP increases PRL release from the primary cultures of decidual stromal cells *in vitro* (49), suggesting that PrRP is also a local modulator of decidual PRL release.

The effect of PrRP on PRL release is controversial in mammals, and such a role has not yet been well investigated in non-mammalian vertebrates. Incubation of the tilapia pituitary with mammalian PrRP had no effect on the release of two PRL isoforms, PRL177 or PRL188, although PrRP2 stimulated the release. Interestingly, ICV injection of mammalian PrRP decreases plasma PRL level in chicks (18). Further studies using homologous systems of fish, amphibians, reptiles, and birds are needed.

#### PrRP2

In contrast to PrRP, PrRP2 is recognized as a strong candidate for the specific stimulator of PRL secretion in teleosts. For example, PrRP2 stimulates PRL release from primary cultures of rainbow trout pituitaries (7). In tilapia, PrRP2 also stimulates PRL release *in vitro* from the rostral pars distalis of the pituitary (8) equipotently with GnRH, another candidate PRL-releasing factor. The PRL-releasing effect of PrRP2 is also observed *in vivo*: plasma PRL concentration increases with intra-arterial (10) and intraperitoneal (IP) injection of PrRP2 (7). In tilapia, injection of PrRP2 elevates plasma PRL levels only in females but not in males (8), in accordance with the gender-biased effects in rats. Additionally, intra-arterial injection of PrRP2 increases the PRL mRNA level in the pituitary of trout *in vivo* (10, 48). Moreover, PrRP2 antiserum decreases mRNA expression of PRL in goldfish, suggesting that endogenous PrRP2 is essential for PRL expression (11).

PrRP2 is thought to be involved in adapting to new osmotic conditions in euryhaline teleosts. This idea is also supported by PRL having an important role in osmotic regulation in teleosts. PRL mRNA expression in the pituitary increases during adaptation to low osmotic conditions. Similarly, PrRP2 mRNA expression increases under freshwater and terrestrial conditions in the brain of euryhaline mudskippers and under ion-poor freshwater in the hypothalamus of goldfish (45). As brain PrRP2, pituitary and gut PrRP2 are also related to acclimation to low osmotic conditions because PrRP2 mRNA in the pituitary and gut increases when euryhaline silver sea bream and mudskipper, respectively, adapt to low salinities (14, 74, 75). These data suggest that PRL expression is regulated by not only brain PrRP but also peripheral PrRP2 in teleosts. In fact, the changes in PRL mRNA in the gut parallel those in PrRP2 mRNA in the gut of mudskipper (74). In addition, the changes in PrRP2 mRNA in the hypothalamus do not parallel that of PRL mRNA in the pituitary (14, 75). These PrRP2 mRNA expressions during osmotic acclimation might be regulated by the olfactory system because removing the olfactory rosette lowers the increase in PrRP2 mRNA expression under hypo-osmotic conditions in sea bream (75).

On the other hand, from bullfrog pituitary cells, PrRP2-20 and PrRP2-31 increased PRL release (to 130–160% of control) *in vitro*, but their effects were much less potent than thyrotropin-releasing hormone-induced PRL release (16). In chicks, similarly, IP injection of PrRP2-31 increased plasma PRL concentration, but the effect was not marked [to 140% of control, (15)]. In contrast, ICV injection of PrRP2-31 decreases plasma PRL level in chicks (15), but the mechanism of this inhibition of PRL release is unclear.

### Effect on hypothalamus–pituitary axes

#### CRH–ACTH axis

In the rat brain, PrRP is produced in some A1/A2 noradrenergic neurons in the medulla oblongata, which mediate stress signals in the central nervous system (76–80). In addition, it has been demonstrated that PrRP neurons innervate the CRH neurons by synaptic connection in rats (37). Stress information sent by A1/A2 noradrenergic neurons activates the hypothalamic CRH neurons, and then stimulates ACTH release from the anterior pituitary (81–83), suggesting that PrRP stimulates ACTH secretion via activating CRH neurons in rodents. Indeed, ICV injection of PrRP increases c-Fos expression in the CRH neurons and plasma ACTH level while systemic injection had no effect (37, 84). Additionally, PrRP-induced ACTH release is blocked by peripheral pretreatment with the CRH antagonist, alpha-helical CRH (37), demonstrating that PrRP activates the CRH neurons for the hypothalamic–pituitary–adrenal (HPA) axis in the stress response. A similar effect of PrRP was also observed in castrated bulls because ICV injection of PrRP increases cortisol secretion (60). In sheep, on the other hand, ICV injection of PrRP does not alter cortisol secretion (62), suggesting that the effect of PrRP is different between animal species. It has been reported that vasopressin rather than CRH might be a major secretagogue of ACTH in sheep, which is different from rats and pigs (85–87). Therefore, the difference in the hypothalamic regulation of the HPA axis might contribute to the unique effect of PrRP in sheep (60).

Intracerebroventricular injection of mammalian PrRP increases the plasma corticosterone level in chicks (18), suggesting that brain PrRP-induced activation of the HPA axis is conserved. On the other hand, the plasma corticosterone level is not changed by ICV injection of PrRP2-20 in chicks (18). In addition, plasma cortisol levels are not increased by IP injection of PrRP2 in tilapia and goldfish (8, 11). These results suggest that PrRP2 might not activate the HPA axis in chicks and teleosts.

#### Somatostatin–GH axis

In rats, somatostatin neurons in the hypothalamic PerVN are contacted by PrRP nerve terminals and express the PrRP receptor. These facts suggest that PrRP alters the activity of somatostatin neurons. Indeed, ICV injection of PrRP-31 induces expression of the immediate early gene (NGFI-A) in these somatostatin neurons in rats (38). The injection of PrRP-31 decreases the plasma GH level (38, 52), and the effect is abolished by depletion or neutralization of somatostatin in rats (38). Therefore, PrRP directly activates somatostatin neurons to stimulate somatostatin release and then inhibits GH release from the anterior pituitary in rodents.

Although the relationship between PrRP and somatostatin has not been well investigated in non-mammalian vertebrates, ICV injection of mammalian PrRP was reported to reduce the plasma GH level in chicks (18), suggesting that their relationship is conserved in other vertebrates. The decrease in GH level was also observed in chicks after PrRP2-31 was centrally injected (15). IP injection of PrRP2 also decreases the GH level in rainbow trout (7). It is, therefore, possible that PrRP2 is also related to the somatostatin–GH axis in non-mammalian vertebrates. PrRP2 does not affect GH secretion in cultured pituitaries of rainbow trout *in vitro* (7), implying that the inhibitory effect of PrRP2 may also be mediated by the release of somatostatin. Thus, it is likely that the inhibitory effect on GH release is well conserved in vertebrates and between PrRP and PrRP2.

#### Other

In mammals, PrRP is thought to regulate oxytocin release because PrRP neurons have synaptic contact with oxytocin neurons in the PVN (36) and PrRP receptors are present in the oxytocin neurons in rats (33). Indeed, central injection of PrRP (10 nmol/300 g rat) elevates the plasma oxytocin level in conscious rats (88). Yamashita et al. (89) also indicated that oxytocin neurons in the hypothalamus and bed nucleus of the stria terminalis show immunoreactivity of PrRP receptors, and that application of PrRP to isolated supraoptic nuclei facilitates the release of oxytocin. They also demonstrated that vasopressin neurons in the hypothalamus show immunoreactivity of PrRP receptors and that PrRP stimulates vasopressin release from the supraoptic nuclei in rats. Similarly, central injection of PrRP also increases the plasma vasopressin level in rats, although the effect is observed only in females (88). PrRP stimulates release of LH and FSH via a hypothalamic mechanism, such as VIP and galanin in rats (70). PrRP is also related to the ovarian steroid-induced LH surge because it is reduced by ICV injection of PrRP antiserum (90). PrRP gene expression is directly regulated by gonadal steroid hormones because PrRP neurons in the medulla oblongata are co-localized with receptors for estrogen or progesterone (53). In addition, administering estrogen or progesterone to ovariectomized rats induces PrRP mRNA expression in the medulla oblongata, as well as release of PrRP in the medial POA (53, 68, 91).

PrRP2 decreases plasma somatolactin levels in rainbow trout at 30 min after intra-arterial injection of PrRP2 (48). On the other hand, Moriyama et al. (7) demonstrated that PrRP2 increases the plasma somatolactin level at 9 h after IP injection, and stimulates somatolactin release only by pharmacological doses of PrRP *in vitro*. The effect of PrRP on somatolactin release might be mediated by an indirect endocrine mechanism as suggested by Moriyama et al. (7), although the distribution of PrRP neuronal terminals are also located near somatolactin cells in the trout pituitary.

### Regulation of food intake and energy homeostasis

Central injection of PrRP, especially into the DMN, reduces food intake in rats without inducing conditioned taste aversion (58, 92–94). The anorexigenic effect is also observed in mice (63). In addition, PrRP mRNA expression decreases with fasting during states of negative energy balance, like other anorexigens, in rats (58). Furthermore, immuno-neutralization of endogenous PrRP using monoclonal antibody has been demonstrated to induce hyperphagia in mice (95). Moreover, hyperphagia is also observed in PrRP-deficient mice (95) and PrRP-R-deficient mice (100). These results show that endogenous PrRP is one of the anorexigenic peptides in mammals. Takayanagi et al. (95) also reveal that PrRP regulates meal size rather than meal frequency in mice. It has been demonstrated that meal size is regulated by several satiety signals including cholecystokin (CCK). CCK is released from the intestine and then activates afferent vagal nerves that project to the medulla oblongata. CCK is also thought to activate PrRP neurons because injection of CCK induces c-Fos expression in PrRP-containing neurons distributed in the NTS (93). In addition, the anorexigenic effect of CCK disappears in both PrRP-deficient mice (95) and PrRP-R-deficient mice (63). Thus, PrRP also appears to mediate the anorexigenic effect of CCK. Leptin is also considered an upstream regulator of the anorexigenic effect of PrRP because the leptin receptor is expressed in PrRP neurons in the brainstem and hypothalamus (94, 96). In addition, PrRP mRNA expression is low in Zucker rats, which are obese due to the lack of a leptin receptor (94). Furthermore, leptin induces the expression of the phosphorylated signal transducer and activator of transcriptional protein 3 in PrRP neurons (95). Moreover, leptin-induced anorexia is not observed in PrRP-deficient mice. Therefore, PrRP also mediates the anorexigenic signal of leptin (95).

The downstream mechanisms underlying the anorexigenic effect of PrRP remain to be clarified. Seal et al. (92) suggested that the anorexigenic effect of PrRP is also mediated by alpha-melanocyte-stimulating hormone (MSH) and neurotensin, both of which are well-known anorexigenic peptides. CRH and oxytocin are also thought to be related to the anorexigenic mechanism of PrRP. As noted above, central injection of PrRP activates CRH or oxytocin neurons in the hypothalamus, and the anorexigenic effect of PrRP is attenuated by a CRH receptor antagonist or oxytocin receptor antagonist (63, 97), showing that they mediate PrRP-induced anorexia. Furthermore, an oxytocin receptor antagonist attenuates the anorexigenic effect of CCK (98, 99). Re-feeding and CCK-induced Fos expression in several brain regions including the PVN was impaired in PrRP-deficient mice. CCK-induced oxytocin release was also impaired in PrRP-deficient mice. Collectively, it is expected that the CCK-PrRP-oxytocin system is an important anorexigenic pathway of PrRP.

Endogenous PrRP is also related to energy metabolism in mammals because PrRP-deficient or PrRP-R-deficient mice show late-onset obesity (95, 100–102). Furthermore, the Otsuka Long-Evans Tokushima Fatty (OLETF) rat strain, which exhibits obesity and diabetes, has a mutated GPR10 gene (103). Streptozotocin-induced diabetic rats also show lower mRNA expression of PrRP and it is reversed by insulin (96). In rats, ICV injection of PrRP increases body temperature and oxygen consumption, also suggesting that brain PrRP is related to the control of energy metabolism (58, 97). In addition to obesity, PrRP-deficient mice show increased food intake, body fat mass, glucose tolerance, and increased levels of blood insulin, leptin, cholesterol, and triacylglycerol (95, 100, 102). Similar changes were also observed in PrRP-R-deficient mice (100, 101). However, PrRP-deficient mice do not show any change in body temperature, oxygen consumption, or locomotion activity (95). PrRP-R-deficient male mice also show no difference in oxygen consumption while consumption is slightly low in PrRP-R-deficient females (101). The PrRP-R-deficient mice become obese but the extent is pronounced in females (101). Thus, it is likely that the role of PrRP in energy metabolism is different between the sexes. The difference is explained by estrogen because PrRP-containing neurons in the brainstem express the estrogen receptor (53). Obesity observed in PrRP-deficient mice is thought to be related to hyperphagia because pair-feeding abolishes the induction of obesity in PrRP-deficient mice (95).

PrRP2 is also recognized as an anorexigenic peptide in teleosts because ICV injection of PrRP2 significantly inhibits feeding behavior in goldfish (45). In mudskipper, a euryhaline fish, brain PrRP2 mRNA expression is induced when they are kept in fresh water (48) where their food intake decreases compared with rearing in sea water (our unpublished observation). Indeed, several euryhaline fish grow slower in fresh water than in seawater (104), suggesting the involvement of brain PrRP2.

In contrast to rodents and teleosts, PrRP shows a unique effect on feeding behavior in steers and chicks. In steers, ICV injection of PrRP has no effect on food intake (61). Furthermore, ICV injection of mammalian PrRP increases food intake in chicks (57). The orexigenic effect is also observed when chicken PrRP is ICV injected (our unpublished data). Similarly, ICV injection of PrRP2-31 also stimulates feeding behavior in chicks, although PrRP2-20 has no effect (15). Thus, the effects of both PrRP and PrRP2 on feeding behavior are completely opposite to those in rodents and teleosts, and seem to have changed during the process of evolution. The reason why PrRP and PrRP2 stimulate feeding behavior in chicks has not yet been well clarified. Interestingly, ICV injection of leptin (105) has no effect on feeding behavior in chicks (105), although CCK, CRH, MSH, and mesotocin (avian homolog of oxytocin) have an anorexigenic effect in chicks (106–109) as well as rodents. The difference in the feeding inhibitory mechanism may contribute to the reason why PrRP does not suppress feeding behavior in chicks. It is possible that NPY, an orexigenic factor, is related to the orexigenic effect of PrRP because ICV injection of PrRP2-31 significantly increases the NPY mRNA level in the diencephalon in chicks (110). As well as in feeding regulation, PrRP2 might also be related to energy metabolism in non-mammalian vertebrates because ICV injection of PrRP2-31 decreases blood insulin, glucose, and non-esterified fatty acid in chicks (110).

### Stress response

As noted in Section “CRH–ACTH Axis,” PrRP neurons project directly to CRH neurons and oxytocin neurons in the hypothalamus (33), and ICV injection of PrRP increases ACTH (84) and oxytocin release in rats (88). These facts suggest that PrRP is a mediator of the HPA axis for stress response. In fact, water immersion-restraint stress dramatically induces c-Fos expression in the A1/A2 neurons containing PrRP (84). Other stress stimuli, such as restraint, conditioned fear, foot shocks, hemorrhage, exercise, and inflammatory stress, also activate PrRP neurons in the medulla oblongata and/or DMN (111–114). Additionally, Seal et al. (92) found that ICV injection of PrRP-31 increases grooming associated with stress response. Immuno-neutralization of PrRP decreases the activation of the neurons in the hypothalamic PVN after noxious stimuli (113) or oxytocin release in response to conditioned fear (111). In addition, contextual conditioned fear-induced oxytocin and ACTH releases are impaired in PrRP-deficient mice (115). It is, therefore, likely that brain PrRP has a facilitative role in stress responses.

In contrast, the injection of PrRP antibodies facilitates ACTH release in response to exercise, suggesting that PrRP inhibits ACTH release in response to exercise (116). Restraint stress-induced corticosterone release is enhanced in PrRP-deficient mice (102). These data suggest that PrRP also inhibits neuroendocrine responses to stress. Additionally, contextual conditioned fear-induced neuroendocrine responses disappear in PrRP-deficient mice while the freezing behavior is augmented (115). The mechanisms underlying these contradictions have not been clarified to date. Takayanagi and Onaka (55) suggest that the role of PrRP in stress responses may depend on the nature of the stressful stimuli used. Thus, PrRP is involved in integration in the control of stress responses, whereas the underlying detailed mechanisms need further investigation.

Intracerebroventricular injection of mammalian PrRP significantly increases the plasma corticosterone level in chicks, implying that brain PrRP is also related to stress response (18). On the other hand, ICV injection of chicken PrRP2-20 has no effect on the plasma corticosterone level, suggesting that PrRP2 might not be involved in stress (18). It is also reported that IP injection of PrRP2 does not alter the plasma cortisol level in tilapia (8). It is, therefore, possible that PrRP2 is not related to activating the HPA axis and stress in non-mammalian vertebrates.

## Concluding Remarks

Prolactin-releasing peptide was originally identified as a stimulator of PRL release in mammals. Although further work revealed that PrRP had less effect on PRL release, PrRP has been shown to be involved in many physiological actions, such as reproduction, endocrine functions, feeding behavior and metabolism, and stress response in mammals, and possibly in other vertebrates (Table 2). This PrRP may be the less appropriate nomenclature, at least for mammals. At the same time, as the discovery of PrRP, PrRP2, whose amino acid sequence is similar to PrRP, was identified from Japanese crucian carp. PrRP and PrRP2 are thought to have originated from a common ancestral gene by the second round of whole genome duplication. In contrast to PrRP, PrRP2 is thought to exist only in non-mammalian vertebrates, and to be essential for PRL secretion in teleosts and important for non-mammalian vertebrates. The physiological actions of PrRP and PrRP2 seem to overlap in non-mammalian vertebrates (Table 2), and may have converged into those of PrRP in mammals. Consequently, the PrRP2 gene might have diminished in mammals during evolution.

The origin of PrRP and PRL might provide a key to knowing how PrRP acquired its diverse functions in vertebrates. Moriyama et al. (17) found two RF-amide peptides (RFa-A and RFa-B), homologous to PrRP, from sea lamprey. The amino acid sequence is similar to PrRP2 rather than PrRP (Figures 1 and 2), especially in RFa-B. If lamprey RFa-A and RFa-B are, respectively, orthologous to PrRP and PrRP2, these peptides originated at least from the stage of primitive vertebrates. Interestingly, lamprey is thought not to possess PRL (117), suggesting that PrRP is generated prior to the occurrence of PRL. Especially in teleosts, PRL is widely expressed in extrapituitary organs, and PrRP2 and its receptor system might concomitantly obtain to regulate these PRL expressions. Conversely, the lack of this extrapituitary PRL might be associated with the fact that the PrRP2-PrRP2 receptor system was not necessary for regulating PRL and disappeared in mammals.

On the other hand, the effect of PrRP on GH secretion is commonly observed among vertebrates. Moriyama et al. (17) found that both RFa-A and RFa-B decrease GH mRNA expression *in vitro* in lamprey. As noted above, PrRP or PrRP2 inhibits GH release from the pituitary in mammals, chicks, and teleosts, suggesting that the roles of PrRP and PrRP2, at least the suppression of GH release, occur with the appearance of primitive vertebrates. Since PRL is a member of the GH family and originated from a common ancestor, the diversity of the physiological roles of PRL and PrRP are thought to have developed with the evolution of GH rather than PRL.

The conserved effect of PrRP on feeding behavior may also be generated in ancestral PrRP. Thus, the original role of PrRP and PrRP2 would be the regulation of GH release and feeding rather than PRL release. However, the physiological roles of PrRP on the releases of PRL and GH, feeding behavior, and stress response in non-mammalian vertebrates have not been clarified (Table 2) because their PrRPs were recently predicted. Information regarding the effect of PrRP2 on the endocrine system and stress response, which have been investigated in rodents, is also insufficient. To acquire a clearer understanding of the biological and physiological roles of PrRP and PrRP2, future investigations should focus on the physiological roles of PrRP and PrRP2 in non-mammalian vertebrates. Such studies should reveal the evolution of the PrRP family in vertebrates.

## Conflict of Interest Statement

The authors declare that the research was conducted in the absence of any commercial or financial relationships that could be construed as a potential conflict of interest.

## References

[1] Hinuma S Habata Y Fujii R Kawamata Y Hosoya M Fukusumi S A prolactin-releasing peptide in the brain. Nature (1998) 393:272–6 10.1038/30515 9607765

[2] Fujimoto M Takeshita K Wang X Takabatake I Fujisawa Y Teranishi H Isolation and characterization of a novel bioactive peptide, *Carassius* RF-amide (C-RFa), from the brain of the Japanese crucian carp. Biochem Biophys Res Commun (1998) 242:436–40. 10.1006/bbrc.1997.7973 9446813

[3] Taylor MM Samson WK. The prolactin releasing peptides: RF-amide peptides. Cell Mol Life Sci (2001) 58:1206–15. 10.1007/PL00000934 11577979 PMC11337406

[4] Lagerström MC Fredriksson R Bjarnadottir TK Schiöth HB. The ancestry of the prolactin-releasing hormone precursor. Ann N Y Acad Sci (2005) 1040:368–70. 10.1196/annals.1327.064 15891064

[5] Wang Y Wang CY Wu Y Huang G Li J Leung FC. Identification of the receptors for prolactin-releasing peptide (PrRP) and *Carassius* RFamide peptide (C-RFa) in chickens. Endocrinology (2012) 153:1861–74. 10.1210/en.2011-1719 22355069

[6] Yamada M Ozawa A Ishii S Shibusawa N Hashida T Ishizuka T Isolation and characterization of the rat prolactin-releasing peptide gene: multiple TATA boxes in the promoter region. Biochem Biophys Res Commun (2001) 281:53–6. 10.1006/bbrc.2001.4308 11178959

[7] Moriyama S Ito T Takahashi T Amano M Sower SA Hirano T A homologue of mammalian prolactin-releasing peptide (fish arginyl-phenylalanyl-amide peptide) is a major hypothalamic peptide of prolactin release in teleost fish. Endocrinology (2002) 143:2071–9. 10.1210/endo.143.6.8744 12021171

[8] Seale AP Itoh T Moriyama S Takahashi A Kawauchi H Sakamoto T Isolation and characterization of a homologue of mammalian prolactin-releasing peptide from the tilapia brain and its effect on prolactin release from the tilapia pituitary. Gen Comp Endocrinol (2002) 125:328–39. 10.1006/gcen.2001.7727 11884078

[9] Montefusco-Siegmund RA Romero A Kausel G Muller M Fujimoto M Figueroa J. Cloning of the prepro C-RFa gene and brain localization of the active peptide in *Salmo salar*. Cell Tissue Res (2006) 325:277–85. 10.1007/s00441-006-0168-6 16557384

[10] Sakamoto T Agustsson T Moriyama S Itoh T Takahashi A Kawauchi H Intra-arterial injection of prolactin-releasing peptide elevates prolactin gene expression and plasma prolactin levels in rainbow trout. J Comp Physiol B (2003) 173:333–7. 10.1007/s00360-003-0340-1 12687398

[11] Fujimoto M Sakamoto T Kanetoh T Osaka M Moriyama S. Prolactin-releasing peptide is essential to maintain the prolactin level and osmotic balance in freshwater teleost fish. Peptides (2006) 27:1104–9. 10.1016/j.peptides.2005.06.034 16519960

[12] Sakamoto T Fujimoto M Ando M. Fishy tales of prolactin-releasing peptide. Int Rev Cytol (2003) 225:91–130. 10.1016/S0074-7696(05)25003-9 12696591

[13] Lagerström MC Fredriksson R Bjarnadóttir TK Fridmanis D Holmquist T Andersson J Origin of the prolactin-releasing hormone (PRLH) receptors: evidence of coevolution between PRLH and a redundant neuropeptide Y receptor during vertebrate evolution. Genomics (2005) 85:688–703. 10.1016/j.ygeno.2005.02.007 15885496

[14] Kwong AKY Woo NYS. Prolactin-releasing peptide, a possible modulator of prolactin in the euryhaline silver sea bream (*Sparus sarba*): a molecular study. Gen Comp Endocrinol (2008) 158:154–60. 10.1016/j.ygcen.2008.06.006 18640118

[15] Tachibana T Moriyama S Takahashi A Tsukada A Oda A Takeuchi S Isolation and characterisation of prolactin-releasing peptide in chicks and its effect on prolactin release and feeding behaviour. J Neuroendocrinol (2011) 23:74–81. 10.1111/j.1365-2826.2010.02078.x 21083629

[16] Sakamoto T Oda A Yamamoto K Kaneko M Kikuyama S Nishikawa A Molecular cloning and functional characterization of a prolactin-releasing peptide homolog from *Xenopus laevis*. Peptides (2006) 27:3347–51. 10.1016/j.peptides.2006.08.003 16979799

[17] Moriyama S Kasahara M Amiya N Takahashi A Amano M Sower SA RFamide peptides inhibit the expression of melanotropin and growth hormone genes in the pituitary of an Agnathan, the sea lamprey, *Petromyzon marinus*. Endocrinology (2007) 148:3740–9. 10.1210/en.2007-0356 17494999

[18] Tachibana T Tsukada A Fujimoto M Takahashi H Ohkubo T Boswell T Comparison of mammalian prolactin-releasing peptide and *Carassius* RFamide for feeding behavior and prolactin secretion in chicks. Gen Comp Endocrinol (2005) 144:264–9. 10.1016/j.ygcen.2005.06.012 16112673

[19] Marchese A Heiber M Nguyen T Heng HH Saldivia VR Cheng R Cloning and chromosomal mapping of three novel genes, GPR9, GPR10, and GPR14, encoding receptors related to interleukin 8, neuropeptide Y, and soma-tostatin receptors. Genomics (1995) 29:335–44. 10.1006/geno.1995.9996 8666380

[20] Welch SK O’Hara BF Kilduff TS Heller HC. Sequence and tissue distribution of a candidate G-coupled receptor cloned from rat hypothalamus. Biochem Biophys Res Commun (1995) 209:606–13. 10.1006/bbrc.1995.1543 7733930

[21] Engström M Brandt A Wurster S Savola JM Panula P. Prolactin releasing peptide has high affinity and efficacy at neuropeptide FF2 receptors. J Pharmacol Exp Ther (2003) 305:825–32. 10.1124/jpet.102.047118 12606605

[22] Roland BL Sutton SW Wilson SJ Luo L Pyati J Huvar R Anatomical distribution of prolactin-releasing peptide and its receptor suggests additional functions in the central nervous system and periphery. Endocrinology (1999) 140:5736–45. 10.1210/endo.140.12.7211 10579339

[23] Langmead CJ Szekeres PG Chambers JK Ratcliffe SJ Jones DN Hirst WD Characterization of the binding of [(125)I]-human prolactin releasing peptide (PrRP) to GPR10, a novel G protein coupled receptor. Br J Pharmacol (2000) 131:683–8. 10.1038/sj.bjp.0703617 11030716 PMC1572376

[24] Satoh F Smith DM Gardiner JV Mahmoodi M Murphy KG Ghatei MA Characterization and distribution of prolactin releasing peptide (PrRP) binding sites in the rat-evidence for a novel binding site subtype in cardiac and skeletal muscle. Br J Pharmacol (2000) 129:1787–93. 10.1038/sj.bjp.0703266 10780987 PMC1572020

[25] Ozawa A Yamada M Satoh T Monden T Hashimoto K Kohga H Transcriptional regulation of the human PRL-releasing peptide (PrRP) receptor gene by a dopamine 2 receptor agonist: cloning and characterization of the human PrRP receptor gene and its promoter region. Mol Endocrinol (2002) 16:785–98. 10.1210/mend.16.4.0819 11923475

[26] Kimura A Ohmichi M Tasaka K Kanda Y Ikegami H Hayakawa J Prolactin-releasing peptide activation of the prolactin promoter is differentially mediated by extracellular signal-regulated protein kinase and c-Jun N-terminal protein kinase. J Biol Chem (2000) 275:3667–74. 10.1074/jbc.275.5.3667 10652364

[27] Hayakawa J Ohmichi M Tasaka K Kanda Y Adachi K Nishio Y Regulation of the PRL promoter by Akt through cAMP response element binding protein. Endocrinology (2001) 143:13–22. 10.1210/endo.143.1.8586 11751586

[28] Duval DL Gutierrez-Hartmann A PRL-releasing peptide stimulation of PRL gene transcription – enter AKT. Endocrinology (2002) 143:11–2 10.1210/endo.143.1.8647 11751585

[29] Watanabe S Kaneko T. Prolactin-releasing peptide receptor expressed in the pituitary in Mozambique tilapia *Oreochromis mossambicus*: an aspect of prolactin regulatory mechanisms. Gen Comp Endocrinol (2010) 167:27–34. 10.1016/j.ygcen.2010.03.004 20226787

[30] Wang X Satake H Sakamoto T Hinuma S Matsumoto H Minakata H A candidate of fish prolactin-releasing peptide isolated from Japanese crucian carp. In: Yu J, editor. Proceedings of 4th Congress of the Asia and Oceania Society for Comparative Endocrinology Taipei (2001). p. 29–38.

[31] Iijima N Kataoka Y Kakihara K Bamba H Tamada Y Hayashi S Cytochemical study of prolactin-releasing peptide (PrRP) in the rat brain. Neuroreport (1999) 10:1713–6. 10.1097/00001756-199906030-00016 10501562

[32] Minami S Nakata T Tokita R Onodera H Imaki J. Cellular localization of prolactin-releasing peptide messenger RNA in the rat brain. Neurosci Lett (1999) 266:73–5. 10.1016/S0304-3940(99)00263-3 10336187

[33] Ibata Y Iijima N Kataoka Y Kakihara K Tanaka M Hosoya M Morphological survey of prolactin-releasing peptide and its receptor with special reference to their functional roles in the brain. Neurosci Res (2000) 38:223–30. 10.1016/S0168-0102(00)00182-6 11070188

[34] Chen C Dun SL Dun NJ Chang JK. Prolactin-releasing peptide-immunoreactive in A1 and A2 noradrenergic neurons of the rat medulla. Brain Res (1999) 822:276–9. 10.1016/S0006-8993(99)01153-1 10082910

[35] Morales T Hinuma S Sawchenko PE. Prolactin-releasing peptide is expressed in afferents to the endocrine hypothalamus, but not in neurosecretory neurones. J Neuroendocrinol (2000) 12:131–40. 10.1046/j.1365-2826.2000.00428.x 10718908

[36] Maruyama M Matsumoto H Fujiwara K Kitada C Hinuma S Onda H Immunocytochemical localization of prolactin-releasing peptide in the rat brain. Endocrinology (1999) 140:2326–33. 10.1210/endo.140.5.6685 10218986

[37] Matsumoto H Maruyama M Noguchi J Horikoshi Y Fujiwara K Kitada C Stimulation of corticotropin-releasing hormone-mediated adrenocorticotropin secretion by central administration of prolactin-releasing peptide in rats. Neurosci Lett (2000) 285:234–8. 10.1016/S0304-3940(00)01077-6 10806329

[38] Iijima N Matsumoto Y Yano T Tanaka M Yamamoto T Kakihara K A novel function of prolactin-releasing peptide in the control of growth hormone via secretion of somatostatin from the hypothalamus. Endocrinology (2001) 142:3239–43. 10.1210/en.142.7.3239 11416047

[39] Yuan ZF Yang SC Pan JT. Effects of prolactin-releasing peptide on tuberoinfundibular dopaminergic neuronal activity and prolactin secretion in estrogen-treated female rats. J Biomed Sci (2002) 9:112–8. 10.1007/BF02256021 11914577

[40] Yamakawa K Kudo K Kanba S Arita J. Distribution of prolactin-releasing peptide-immunoreactive neurons in the rat hypothalamus. Neurosci Lett (1999) 267:113–6. 10.1016/S0304-3940(99)00346-8 10400225

[41] Fujii R Fukusumi S Hosoya M Kawamata Y Habata Y Hinuma S Tissue distribution of prolactin-releasing peptide (PrRP) and its receptor. Regul Pept (1999) 83:1–10 10.1016/S0167-0115(99)00028-2 10498338

[42] Takahashi K Yoshinoya A Arihara Z Murakami O Totsune K Sone M Regional distribution of immunoreactive prolactin-releasing peptide in the human brain. Peptides (2000) 21:1551–5. 10.1016/S0196-9781(00)00310-7 11068103

[43] Curlewis JD Kusters DH Barclay JL Anderson ST. Prolactin-releasing peptide in the ewe: cDNA cloning, mRNA distribution and effects on prolactin secretion in vitro and in vivo. J Endocrinol (2002) 174:45–53. 10.1677/joe.0.1740045 12098662

[44] Matsumoto H Murakami Y Horikoshi Y Noguchi J Habata Y Kitada C Distribution and characterization of immunoreactive prolactin-releasing peptide (PrRP) in rat tissue and plasma. Biochem Biophys Res Commun (1999) 257:264–8. 10.1006/bbrc.1999.0463 10198200

[45] Kelly SP Peter RE. Prolactin-releasing peptide, food intake, and hydromineral balance in goldfish. Am J Physiol Regul Integr Comp Physiol (2006) 291:R1474–81. 10.1152/ajpregu.00129.2006 16741144

[46] Satake H Minakata H Wang X Fujimoto M. Characterization of a cDNA encoding a precursor of *Carassius* RFamide, structurally related to a mammalian prolactin-releasing peptide. FEBS Lett (1999) 446:247–50. 10.1016/S0014-5793(99)00215-X 10100851

[47] Wang X Morishita F Matsushima O Fujimoto M. Immunohistochemical localization of C-RFamide, a FMRF-related peptide, in the brain of the goldfish, *Carassius auratus*. Zoolog Sci (2000) 17:1067–74. 10.2108/zsj.17.1067 18522460

[48] Sakamoto T Iwata K Ando M Growth hormone and prolactin expression during environmental adaptation of gobies. Fish Sci (2002) 68:757–60 10.2331/fishsci.68.sup1_757

[49] Reis FM Vigano P Arnaboldi E Spritzer PM Petraglia F Di Blasio AM. Expression of prolactin-releasing peptide and its receptor in the human decidua. Mol Hum Reprod (2002) 8:356–62. 10.1093/molehr/8.4.356 11912284

[50] Samson WK Resch ZT Murphy TC. A novel action of the newly described prolactin-releasing peptides: cardiovascular regulation. Brain Res (2000) 858:19–25. 10.1016/S0006-8993(99)02451-8 10700591

[51] Wang X Morishita F Matsushima O Fujimoto M. *Carassius* RFamide, a novel FMRFa-related peptide, is produced within the retina and involved in retinal information processing in cyprinid fish. Neurosci Lett (2000) 289:115–8. 10.1016/S0304-3940(00)01281-7 10904133

[52] Zhang SQ Inoue S Kimura M. Sleep-promoting activity of prolactin-releasing peptide (PrRP) in the rat. Neuroreport (2001) 12:3173–6. 10.1097/00001756-200110290-00006 11711850

[53] Kataoka Y Iijima N Yano T Kakihara K Hayashi S Hinuma S Gonadal regulation of PrRP mRNA expression in the nucleus tractus solitarius and ventral and lateral reticular nuclei of the rat. Brain Res Mol Brain Res (2001) 87:42–7. 10.1016/S0169-328X(00)00280-1 11223158

[54] Sun B Fujiwara K Adachi S Inoue K Physiological roles of prolactin-releasing peptide. Regul Pept (2005) 126:27–33 10.1016/j.regpep.2004.08.008 15620410

[55] Takayanagi Y Onaka T. Roles of prolactin-releasing peptide and RFamide related peptides in the control of stress and food intake. FEBS J (2010) 277:4998–5005. 10.1111/j.1742-4658.2010.07932.x 21126313

[56] Dodd GT Luckman SM. Physiological roles of GPR10 and PrRP signaling. Front Endocrinol (2013) 4:20. 10.3389/fendo.2013.00020 23467899 PMC3587801

[57] Tachibana T Saito S Tomonaga S Takagi T Saito ES Nakanishi T Effect of central administration of prolactin-releasing peptide on feeding in chicks. Physiol Behav (2004) 80:713–9. 10.1016/j.physbeh.2003.12.005 14984806

[58] Lawrence CB Celsi F Brennand J Luckman SM. Alternative role for prolactin-releasing peptide in the regulation of food intake. Nat Neurosci (2000) 3:645–6. 10.1038/76597 10862694

[59] Rubinek T Hadani M Barkai G Melmed S Shimon I. Prolactin (PRL)-releasing peptide stimulates PRL secretion from human fetal pituitary cultures and growth hormone release from cultured pituitary adenomas. J Clin Endocrinol Metab (2001) 86:2826–30. 10.1210/jcem.86.6.7591 11397894

[60] Kitagawa S Abe N Sutoh M Kasuya E Sugita S Aoyama M Effect of intracerebroventricular injections of prolactin-releasing peptide on prolactin release and stress-related responses in steers. Anim Sci J (2011) 82:314–9. 10.1111/j.1740-0929.2010.00841.x 21729212

[61] Yayou K Kitagawa S Ito S Kasuya E Sutoh M Effect of oxytocin, prolactin-releasing peptide, or corticotropin-releasing hormone on feeding behavior in steers. Gen Comp Endocrinol (2011) 174:287–91 10.1016/j.ygcen.2011.09.003 21945119

[62] Mogi K Ito S Matsuyama S Ohara H Sakumoto R Yayou K Central administration of neuropeptide B, but not prolactin-releasing peptide, stimulates cortisol secretion in sheep. J Reprod Dev (2008) 54:138–41. 10.1262/jrd.19109 18239355

[63] Bechtold DA Luckman SM. Prolactin-releasing peptide mediates cholecystokinin-induced satiety in mice. Endocrinology (2006) 147:4723–9. 10.1210/en.2006-0753 16794001

[64] Ben-Jonathan N Arbogast LA Hyde JF Neuroendocrine [corrected] regulation of prolactin release. Prog Neurobiol (1989) 33:399–447 10.1016/0301-0082(89)90008-7 2695976

[65] Samson WK Resch ZT Murphy TC Chang JK. Gender-biased activity of the novel prolactin releasing peptides: comparison with thyrotropin releasing hormone reveals only pharmacologic effects. Endocrine (1998) 9:289–91. 10.1385/ENDO:9:3:289 10221595

[66] Kawamata Y Fujii R Fukusumi S Habata Y Hosoya M Hinuma S Analyses for susceptibility of rat anterior pituitary cells to prolactin-releasing peptide. Endocrine (2000) 12:215–21. 10.1385/ENDO:12:3:215 10963040

[67] Matsumoto H Noguchi J Horikoshi Y Kawamata Y Kitada C Hinuma S Stimulation of prolactin release by prolactin-releasing peptide in rats. Biochem Biophys Res Commun (1999) 259:321–4. 10.1006/bbrc.1999.0789 10362506

[68] Tokita R Nakata T Katsumata H Konishi S Onodera H Imaki J Prolactin secretion in response to prolactin-releasing peptide and the expression of the prolactin-releasing peptide gene in the medulla oblongata are estrogen dependent in rats. Neurosci Lett (1999) 276:103–6. 10.1016/S0304-3940(99)00796-X 10624802

[69] Hashizume T Sasaki T Nonaka S Hayashi T Takisawa M Horiuchi M Bovine posterior pituitary extract stimulates prolactin release from the anterior pituitary gland in vitro and in vivo in cattle. Reprod Domest Anim (2005) 40:184–9 10.1111/j.1439-0531.2005.00580.x 15819972

[70] Seal LJ Small CJ Kim MS Stanley SA Taheri S Ghatei MA Prolactin releasing peptide (PrRP) stimulates luteinizing hormone (LH) and follicle stimulating hormone (FSH) via a hypothalamic mechanism in male rats. Endocrinology (2000) 141:1909–12. 10.1210/endo.141.5.7528 10803604

[71] Watanobe H Schiöth HB Wikberg JE Suda T. Evaluation of the role for prolactin-releasing peptide in prolactin secretion induced by ether stress and suckling in the rat: comparison with vasoactive intestinal peptide. Brain Res (2000) 865:91–6. 10.1016/S0006-8993(00)02164-8 10814736

[72] Zhang SQ Kimura M Inoue S. Effects of prolactin-releasing peptide (PrRP) on sleep regulation in rats. Psychiatry Clin Neurosci (2000) 54:262–4. 10.1046/j.1440-1819.2000.00670.x 11186069

[73] Yuan ZF Pan JT. Involvement of angiotensin II, TRH and prolactin-releasing peptide in the estrogen-induced afternoon prolactin surge in female rats: studies using antisense technology. Life Sci (2002) 71:899–910. 10.1016/S0024-3205(02)01773-3 12084387

[74] Sakamoto T Amano M Hyodo S Moriyama S Takahashi A Kawauchi H Expression of prolactin-releasing peptide and prolactin in the euryhaline mudskippers (*Periophthalmus modestus*): prolactin-releasing peptide as a primary regulator of prolactin. J Mol Endocrinol (2005) 34:825–34. 10.1677/jme.1.01768 15956350

[75] Kwong AKY Woo NYS. The importance of the olfactory rosettes in maintaining pituitary prolactin and prolactin-releasing peptide levels during hyposmotic acclimation in silver sea bream (*Sparus sarba*). Comp Biochem Physiol A Mol Integr Physiol (2012) 161:456–62. 10.1016/j.cbpa.2012.01.005 22266396

[76] Palkovits M Zaborszky L Feminger A Mezey E Fekete MI Herman JP Noradrenergic innervation of the rat hypothalamus: experimental biochemical and electron microscopic studies. Brain Res (1980) 191:161–71. 10.1016/0006-8993(80)90320-0 7378748

[77] Liposits Z Phelix C Paull WK. Adrenergic innervation of corticotropin-releasing factor (CRF)-synthesizing neurons in the hypothalamic paraventricular nucleus of the rat: a combined light and electron microscopic immunocytochemical study. Histochemistry (1986) 84:201–5. 10.1007/BF00495783 3519543

[78] Gaillet S. Effects of discrete lesions in the ventral noradrenergic ascending bundle on the corticotropic stress response depending on the site of the lesion and on the plasma levels of adrenal steroids. Neuroendocrinology (1993) 58:408–19. 10.1159/000126570 8284026

[79] Pacak K Palkovits M Kvetnansky R Kopin IJ Goldstein DS. Stress-induced norepinephrine release in the paraventricular nucleus of rats with brainstem hemisections: a microdialysis study. Neuroendocrinology (1993) 58:196–201. 10.1159/000126533 8264865

[80] Pezzone MA Lee WS Hoffman GE Pezzone KM Rabin BS. Activation of brainstem catecholaminergic neurons by conditioned and unconditioned aversive stimuli as revealed by c-Fos immunoreactivity. Brain Res (1993) 608:310–8. 10.1016/0006-8993(93)91472-5 8495365

[81] Alonso G Szafarczyk A Balmefrezol M Assenmacher I. Immunocytochemical evidence for stimulatory control by the ventral noradrenergic bundle of parvocellular neurons of the paraventricular nucleus secreting corticotropin releasing hormone and vasopressin in rats. Brain Res (1986) 397:297–307. 10.1016/0006-8993(86)90631-1 3099973

[82] Sawchenko PE. Effects of catecholamine-depleting medullary knife cuts on corticotropin-releasing factor and vasopressin immunoreactivity in the hypothalamus of normal and steroid-manipulated rats. Neuroendocrinology (1988) 48:459–70. 10.1159/000125050 2469027

[83] Ito K. Microinjection of norepinephrine into the paraventricular nucleus of the hypothalamus stimulates corticotropin-releasing factor gene expression in conscious rats. Endocrinology (1994) 135:2177–82. 10.1210/endo.135.5.7956940 7956940

[84] Maruyama M Matsumoto H Fujiwara K Noguchi J Kitada C Fujino M Prolactin-releasing peptide as a novel stress mediator in the central nervous system. Endocrinology (2001) 142:2032–8. 10.1210/en.142.5.2032 11316770

[85] Watanabe T Orth DN. Effects of several in vitro systems on the potencies of putative adrenocorticotropin secretagogues on rat anterior pituitary cells. Endocrinology (1988) 122:2299–308. 10.1210/endo-122-5-2299 2834188

[86] Familari M Smith AI Smith R Funder JW. Arginine vasopressin is a much more potent stimulus to ACTH release from ovine anterior pituitary cells than ovine corticotropin-releasing factor. 1. In vitro studies. Neuroendocrinology (1989) 50:152–7. 10.1159/000125214 2550836

[87] Minton JE Parsons KM. Adrenocorticotropic hormone and cortisol response to corticotropin-releasing factor and lysine vasopressin in pigs. J Anim Sci (1993) 71:724–9. 8385088 10.2527/1993.713724x

[88] Maruyama M Matsumoto H Fujiwara K Noguchi J Kitada C Hinuma S Central administration of prolactin-releasing peptide stimulates oxytocin release in rats. Neurosci Lett (1999) 276:193–6. 10.1016/S0304-3940(99)00831-9 10612638

[89] Yamashita M Takayanagi Y Yoshida M Nishimori K Kusama M Onaka T. Involvement of prolactin-releasing peptide in the activation of oxytocin neurones in response to food intake. J Neuroendocrinol (2013) 25:455–65. 10.1111/jne.12019 23363338 PMC3664423

[90] Hizume T Watanobe H Yoneda M Suda T Schiöth HB. Involvement of prolactin-releasing peptide in the preovulatory luteinizing hormone and prolactin surges in the rat. Biochem Biophys Res Commun (2000) 279:35–9. 10.1006/bbrc.2000.3895 11112414

[91] Watanobe H. In vivo release of prolactin-releasing peptide in rat hypothalamus in association with luteinizing hormone and prolactin surges. Neuroendocrinology (2001) 74:359–66. 10.1159/000054702 11752892

[92] Seal LJ Small CJ Dhillo WS Stanley SA Abbott CR Ghatei MA PRL-releasing peptide inhibits food intake in male rats via the dorsomedial hypothalamic nucleus and not the paraventricular hypothalamic nucleus. Endocrinology (2001) 142:4236–43. 10.1210/endo.142.10.8419 11564679

[93] Lawrence CB Ellacott KL Luckman SM. PRL-releasing peptide reduces food intake and may mediate satiety signaling. Endocrinology (2002) 143:360–7. 10.1210/endo.143.2.8609 11796487

[94] Ellacott KL Lawrence CB Rothwell NJ Luckman SM. PRL-releasing peptide interacts with leptin to reduce food intake and body weight. Endocrinology (2002) 143:368–74. 10.1210/endo.143.2.8608 11796488

[95] Takayanagi Y Matsumoto H Nakata M Mera T Fukusumi S Hinuma S Endogenous prolactin-releasing peptide regulates food intake in rodents. J Clin Invest (2008) 118:4014–24. 10.1172/JCI34682 19033670 PMC2575834

[96] Mera T Fujihara H Saito J Kawasaki M Hashimoto H Saito T Downregulation of prolactin-releasing peptide gene expression in the hypothalamus and brainstem of diabetic rats. Peptides (2007) 28:1596–604. 10.1016/j.peptides.2007.06.023 17681402

[97] Lawrence CB Liu YL Stock MJ Luckman SM. Anorectic actions of prolactin-releasing peptide are mediated by corticotropin-releasing hormone receptors. Am J Physiol Regul Integr Comp Physiol (2004) 286:R101–7. 10.1152/ajpregu.00402.2003 14512273

[98] Olson BR Drutarosky MD Stricker EM Verbalis JG. Brain oxytocin receptor antagonism blunts the effects of anorexigenic treatments in rats: evidence for central oxytocin inhibition of food intake. Endocrinology (1991) 129:785–91. 10.1210/endo-129-2-785 1649746

[99] Blevins JE Eakin TJ Murphy JA Schwartz MW Baskin DG. Oxytocin innervation of caudal brainstem nuclei activated by cholecystokinin. Brain Res (2003) 993:30–41. 10.1016/j.brainres.2003.08.036 14642828

[100] Gu W Geddes BJ Zhang C Foley KP Stricker-Krongrad A. The prolactin-releasing peptide receptor (GPR10) regulates body weight homeostasis in mice. J Mol Neurosci (2004) 22:93–103. 10.1385/JMN:22:1-2:93 14742914

[101] Bjursell M Lenneras M Goransson M Elmgren A Bohlooly YM. GPR10 deficiency in mice results in altered energy expenditure and obesity. Biochem Biophys Res Commun (2007) 363:633–8. 10.1016/j.bbrc.2007.09.016 17904108

[102] Mochiduki A Takeda T Kaga S Inoue K. Stress response of prolactin-releasing peptide knockout mice as to glucocorticoid secretion. J Neuroendocrinol (2010) 22:576–84. 10.1111/j.1365-2826.2010.01993.x 20298457

[103] Watanabe TK Suzuki M Yamasaki Y Okuno S Hishigaki H Ono T Mutated G-protein-coupled receptor GPR10 is responsible for the hyperphagia/dyslipidaemia/obesity locus of Dmo1 in the OLETF rat. Clin Exp Pharmacol Physiol (2005) 32:355–66 10.1111/j.1440-1681.2005.04196.x 15854142

[104] Ron B Shimoda SK Iwama GK Grau EG Relationship among ration, salinity, 17α-methyltestosterone and growth in the euryhaline tilapia, *Oreochromis mossambicus*. Aquaculture (1995) 135:185–93 10.1016/0044-8486(95)01013-0

[105] Bungo T Shimojo M Masuda Y Tachibana T Tanaka S Sugahara K Intracerebroventricular administration of mouse leptin does not reduce food intake in the chicken. Brain Res (1999) 817:196–8. 10.1016/S0006-8993(98)01223-2 9889365

[106] Furuse M Matsumoto M Saito N Sugahara K Hasegawa S. The central corticotropin-releasing factor and glucagon-like peptide-1 in food intake of the neonatal chick. Eur J Pharmacol (1997) 339:211–4. 10.1016/S0014-2999(97)01391-5 9473137

[107] Furuse M Ao R Bungo T Ando R Shimojo M Masuda Y Central gastrin inhibits feeding behavior and food passage in neonatal chicks. Life Sci (1999) 65:305–11. 10.1016/S0024-3205(99)00249-0 10447216

[108] Kawakami S Bungo T Ando R Ohgushi A Shimojo M Masuda Y Central administration of alpha-melanocyte stimulating hormone inhibits fasting- and neuropeptide Y-induced feeding in neonatal chicks. Eur J Pharmacol (2000) 398:361–4. 10.1016/S0014-2999(00)00344-7 10862825

[109] Masunari K Khan MS Cline MA Tachibana T. Central administration of mesotocin inhibits feeding behavior in chicks. Regul Pept (2013) 187:1–5. 10.1016/j.regpep.2013.10.004 24183984

[110] Tachibana T Moriyama S Khan MS Sakamoto T. Central administration of prolactin-releasing peptide shifts the utilities of metabolic fuels from carbohydrate to lipids in chicks. Physiol Behav (2013) 120:40–5. 10.1016/j.physbeh.2013.06.017 23816984

[111] Zhu LL Onaka T. Facilitative role of prolactin-releasing peptide neurons in oxytocin cell activation after conditioned-fear stimuli. Neuroscience (2003) 118:1045–53. 10.1016/S0306-4522(03)00059-9 12732249

[112] Onaka T. Neural pathways controlling central and peripheral oxytocin release during stress. J Neuroendocrinol (2004) 16:308–12. 10.1111/j.0953-8194.2004.01186.x 15089967

[113] Mera T Fujihara H Kawasaki M Hashimoto H Saito T Shibata M Prolactin-releasing peptide is a potent mediator of stress responses in the brain through the hypothalamic paraventricular nucleus. Neuroscience (2006) 141:1069–86. 10.1016/j.neuroscience.2006.04.023 16730416

[114] Uchida K Kobayashi D Das G Onaka T Inoue K Itoi K. Participation of the prolactin-releasing peptide-containing neurones in caudal medulla in conveying haemorrhagic stress-induced signals to the paraventricular nucleus of the hypothalamus. J Neuroendocrinol (2010) 22:33–42. 10.1111/j.1365-2826.2009.01935.x 19912474

[115] Yoshida M Takayanagi Y Onaka T. The medial amygdala-medullary PrRP-synthesizing neuron pathway mediates neuroendocrine responses to contextual conditioned fear in male rodents. Endocrinology (2014) 155:2996–3004. 10.1210/en.2013-1411 24877622 PMC4207914

[116] Ohiwa N Chang H Saito T Onaka T Fujikawa T Soya H. Possible inhibitory role of prolactin releasing peptide for ACTH release associated with running stress. Am J Physiol Regul Integr Comp Physiol (2007) 292:R497–504. 10.1152/ajpregu.00345.2006 16917019

[117] Bently PJ The chemical structure, polymorphism, and evolution of hormones. 3rd ed Comparative Vertebrate Endocrinology. Cambridge: Cambridge University Press (1998). p. 65–176.

